# IFN-I-Mediated Transcriptional Reprogramming Drives Myeloid-skewed Hematopoiesis in Sickle Cell Anemia

**DOI:** 10.21203/rs.3.rs-7766748/v1

**Published:** 2025-10-07

**Authors:** Marion Serra, Marine Aglave, Mohammad Salma, Steven Akkaya, Patricia Hermand, Carine Lefevre, Romain Duval, Jade Merrer, Fallou Leye, Joelle Magne, Isabelle Plo, Lionel Blanc, Eric Soler, Slim Azouzi, Berengere Koehl

**Affiliations:** Université Paris Cité, INSERM; Gustave Roussy, Plateforme de bioinformatique; IGMM, Univ Montpellier, CNRS; Université Paris Cité, INSERM; Université Paris Cité, INSERM; Université Paris Cité, INSERM; Université Paris Cité, INSERM, EFS; Unité d’Epidémiologie Clinique, INSERM CIC 1426; Unité d’Epidémiologie Clinique, INSERM CIC 1426; Université Paris Cité, INSERM; Inserm UMR 1287, Université Paris Saclay; Institute of Molecular Medicine, Feinstein Institutes for Medical Research, Northwell; IGMM, Univ Montpellier, CNRS; Université Paris Cité, INSERM, EFS; Sickle Cell Disease Center, Hematology Unit, Hôpital Robert Debré, Assistance Publique – Hôpitaux de Paris

**Keywords:** Sickle Cell Anemia, Inflammation, Myelopoiesis, Interferon

## Abstract

Neutrophils and monocytes are persistently elevated in sickle cell anemia (SCA), yet the intrinsic mechanisms driving pathological myelopoiesis and inflammation remain poorly defined. Through single-cell RNA sequencing and functional assays, we demonstrate that hematopoietic stem and multipotent progenitor cells (HSPCs) in SCA are transcriptionally reprogrammed toward myeloid differentiation. This process is orchestrated by aberrant activation of type I interferon (IFN-I) signaling, which promotes premature myeloid commitment of hematopoietic stem cells. SCA progenitors further exhibit unexpected responsiveness to granulocyte colony-stimulating factor (G-CSF) through upregulation of CSF3R, resulting in skewed myelopoiesis toward the monocytic lineage. Importantly, hydroxyurea treatment attenuates IFN-I signaling in neutrophils, consistent with its therapeutic role in reducing excessive inflammation and granulopoiesis. Collectively, these findings uncover IFN-I–driven remodeling of hematopoiesis as a fundamental mechanism of leukocytosis and chronic inflammation in SCA, and establish a tractable therapeutic axis to mitigate innate immunity activation in this disease.

## Introduction

Sickle cell anemia (SCA) is a severe genetic disorder originating from a single mutation in the β-globin gene, leading to the replacement of the hydrophilic glutamic acid by the hydrophobic valine in the β-globin chain. In deoxygenated conditions, polymerization of the abnormal hemoglobin S (HbS) induces sickling of red blood cells (RBC), and subsequent obstruction of small vessels. SCA is characterized by chronic hemolytic anemia, recurrent vaso-occlusive crisis (VOC), and progressive multiple organ failure^1^. More recently, chronic sterile inflammation has been defined as a hallmark of SCA ^2^, highlighting the crucial role of neutrophils and monocytes in addition to the well-known role of red cells in the pathophysiology of the disease. Neutrophils from patients with SCA present multiple abnormalities. Early studies have reported a higher neutrophil count in patients with SCA at steady state compared to ethnicity-matched healthy donors (HD), and a positive correlation with SCA severity and overall mortality ^3–5^. Conversely, a reduction in the neutrophil count in patients treated with hydroxyurea (HU) can reduce the severity of the disease ^6^. Other neutrophil dysfunctions include overexpression of adhesion molecules, increased formation of neutrophil extracellular traps (NET), and persistence of circulating aged neutrophils ^7–9^. Furthermore, patients with SCA show a high monocyte count at steady state, with an activated phenotype, as indicated by overexpression of adhesion molecules, and increased production of proinflammatory cytokines (IL1β and TNF) ^10,11^. Currently, the origin of hyperleukocytosis in SCA remains elusive. It has been considered a peripheral phenomenon driven by chronic RBC hemolysis, causing high production of pro-inflammatory cytokines and subsequent neutrophil recruitment. Chronic hemolysis could also result in bone marrow (BM) hyperstimulation to replenish RBCs, and simultaneously stimulate leukocyte production ^12,13^. Interestingly, we recently showed that a drastic reduction of hemolysis in patients on chronic transfusion exchange does not improve leukocyte dysfunctions, suggesting that hemolysis might not be the only driver of chronic inflammation in SCA ^14^. In parallel, several reports suggested that the hematopoietic stem and progenitor cell (HSPC) compartment might be impacted in SCA. Indeed, reduced frequency of HSCs in the BM of SCA murine models and patients with SCA have been documented; however, these patients were mainly treated with HU ^15–18^. Furthermore, inflammatory and immune responses were found upregulated in HSPCs from patients with SCA, suggesting that chronic inflammatory state in SCA could disturb HSPCs functions^19^. Chronic inflammation has also been shown to enhance myelopoiesis by skewing HSCs toward the myeloid lineage in different pathological conditions, ultimately resulting in increased circulating mature myeloid cells ^20–23^. Consequently, we made the hypothesis that leukocytosis in SCA might result from hematopoietic dysfunction. Using circulating CD34^+^ cells from HD and untreated patients with SCA, we combined transcriptomic, phenotypic, and functional approaches to reveal abnormal myeloid features of SCA HSPCs. We provide evidence of a skewing of HSPCs towards the myeloid lineage, which is associated with activation of the IFN-I pathway, and the unexpected monocytic differentiation upon *ex vivo* stimulation with G-CSF.

## Methods

Detailed protocols for all methods are provided in supplementary methods.

### Blood samples

Peripheral blood (PB) samples were collected from patients with SCA (homozygous SS) and HDs (Etablissement Français du Sang (EFS)). Patients with SCA were children aged 3 to 17 years old, treated neither with HU nor chronic transfusions, and at a distance from any infection or vaso-occlusive event, having an exchange transfusion as preparation for surgery.

The newborn cohort included 739 children with SCA (homozygotes SS) born between January 1996 and January 2022. They were all diagnosed through newborn screening and followed at Robert Debré Hospital SCD center. Patients receiving treatment with Hydroxyurea before the age of 2 years-old were excluded. Biological data were prospectively entered into a database (Base SDM/Epiconcept-Voozanoo, version 2.0, Epiconcept^™^) as part of standard patient management. Oral information about the database was provided to the parents and/or the patient when adult. Non-opposition was reported on the medical chart. The database was approved by the French National Committee for Computerized Databases (CNIL number 1299665).

The use of the data base and the collection of blood sample for the study was approved by the French Ethical Evaluation Committee for Biomedical Research Projects (CPP n ° 2018-A02894–51). Consent was obtained from all participants prior to inclusion. The study was conducted in accordance with the Declaration of Helsinki.

### Isolation of CD34 cells

CD34^+^ cells were isolated from peripheral blood mononuclear cells (PBMC) using Ficoll density gradient followed by immunomagnetic separation.

### CD34 cells culture

CD34^+^ cells from HDs and patients with SCA at distance of RBC transfusion and never treated with HU were both used for colony forming assay and *ex vivo* granulopoiesis. CFU assays were performed using Methocult^™^ H4435 Enriched medium to evaluate CFU-GEMM, BFU-E, and CFU-GM colonies. For *ex vivo* granulopoiesis, CD34^+^ cells were cultured in presence of SCF, IL3, Flt3L, G-CSF and plasma for 19 days.

### Flow cytometry

Expression of surface markers, cell viability and cell cycle were monitored using fluorochrome-conjugated antibodies or permeable dyes (see supplementary methods).

### Single cell RNA sequencing

Single-cell RNA sequencing (scRNAseq) of CD34^+^ was carried out using the 10x Genomics Chromium system with library preparation and sequencing on Illumina NovaSeq 6000. Data integration and clustering were achieved using Harmony and Seurat ^24,25^, with Uniform Manifold Approximation and Projection (UMAP) for visualization.

### Proteomics of mature neutrophils

Proteomic of neutrophils from HDs and patients with SCA was investigated by Orbitrap Fusion mass spectrometer. Raw data were processed with MaxQuant and the UniProt-SwissProt human database for protein identification and label-free quantification. Downstream statistical analyses were carried out using Perseus software version 1.6.2.3.

## Results

### HbF levels and reticulocyte counts are associated with leukocytosis in SCA

**Figure 1.**
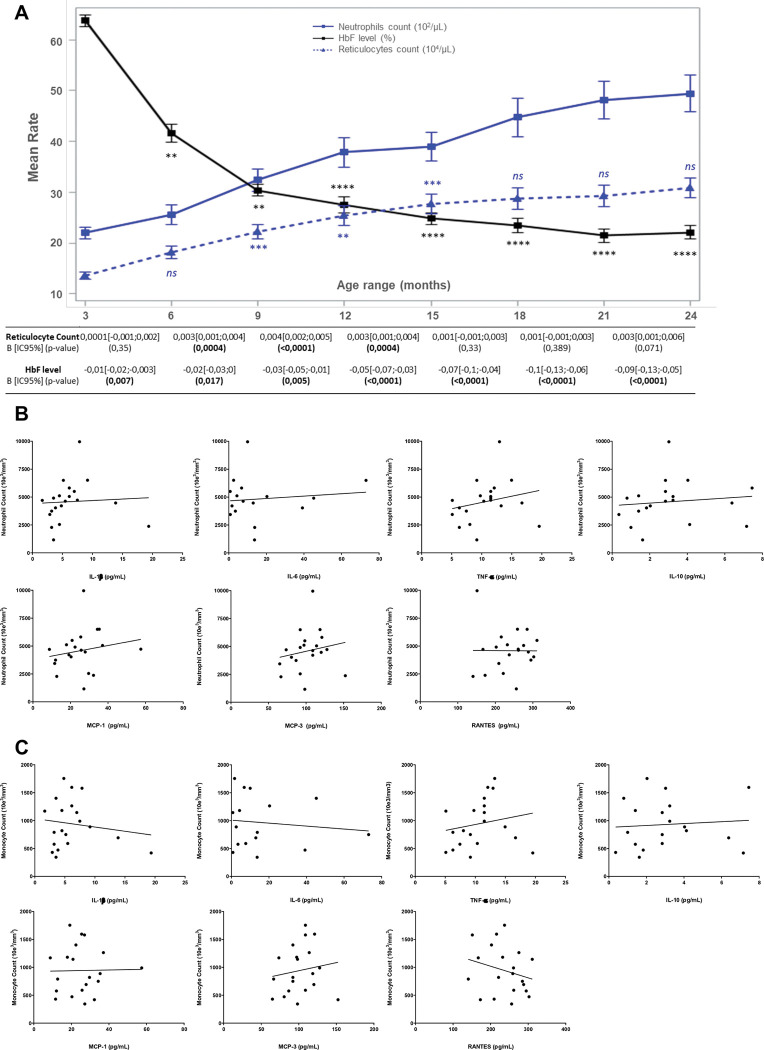
Association of high neutrophil count with hemolysis, HbF switch and pro-inflammatory cytokines in children with SCA. **(A)** Association of neutrophils’ increase with reticulocyte count and HbF level using 1969 longitudinal biological data from 739 children with SCA aged 1–24 months. Graphical representation of the evolution of neutrophils (blue), reticulocytes (dotted blue) and HbF (black) rates during the first two years of life in patients with SCA. Factors correlated with neutrophil count between successive age ranges are also shown. For each age range, the impact of reticulocyte and HbF levels on neutrophil count is assessed with β coefficient [95% confidence interval] and p-value (5% significant threshold). Bold numbers represent significant p-value. **(B)** Plasmatic cytokine level (IL1beta, Il10, Il18, Il6, MCP-1, MCP-3, RANTES and TNFalpha) were measured in the plasma of 20 patients with SCA not receiving hydroxyurea treatment and at steady state. These levels were then compared with the patients’ neutrophil and monocyte counts measured on the same day. No correlation was found between any cytokine levels and neutrophil or monocyte count using a simple linear regression analysis.

To better understand when and how hyperleukocytosis begins in patients with SCA, we analyzed 1969 longitudinal biological data from 739 children with SCA aged 1–24 months. For each patient, 1 to 4 samples collected at different time points during the two first years of life were used. Table S1 displays the evolution of biological parameters (hemoglobin level, HbF percentage, leukocyte, neutrophil, and reticulocyte counts, bilirubin level, and SGOT dosage), showing the onset of high leukocytosis, in conjunction with the drop of HbF level and the increase in hemolysis parameters between 1 and 24 months old. We observed that the neutrophil count increased as early as within the first months of life, rising from 1.8×10^9^/L at 3 months to 4.5×10^9^/L at 24 months (Figure 1A, Table S1). Furthermore, infants with SCA displayed a progressive increase in reticulocyte counts, SGOT and bilirubin levels (hemolysis markers), as well as a physiological decrease in fetal hemoglobin (HbF) levels (Figure 1A, Table S1). Next, we analyzed the association between the increase in neutrophil numbers and the respective changes in reticulocyte count and HbF. Variations in these two clinical parameters were overall significantly correlated with the increase of the neutrophil count, but variations in HbF level had a greater impact on the increase of neutrophil count than variations in reticulocyte count. Specifically, before the age of 15 months, both the reticulocyte count and HbF levels were associated with neutrophil variations, with HbF level having the greatest impact (Figure 1A). Between 15 and 21 months, the increase of reticulocytes was not associated with the progression of the neutrophil count while HbF decrease continued to have a great impact on the onset of hyperleukocytosis (Figure 1A). Given the known role of inflammatory cytokines in SCA pathophysiology, we next examined whether circulating cytokine levels were associated with neutrophil and monocyte counts in children from 2 to 17 years of age. However, none of the tested cytokines (IL-1β, IL-6, TNF-α, IL-10, MCP-1, MCP-3, and RANTES) showed significant correlations with neutrophil or monocyte counts. (Figure 1B-C). Overall, these results balance the hypothesis that neutrophilia is only driven by chronic intravascular hemolysis and suggest that leukocytosis may be related to a dynamic change in hematopoietic progenitor features and involve alternative cytokine pathways.

### ScRNA-seq analysis identifies an enhanced myeloid commitment of HSPCs in SCA.

**Figure 2.**
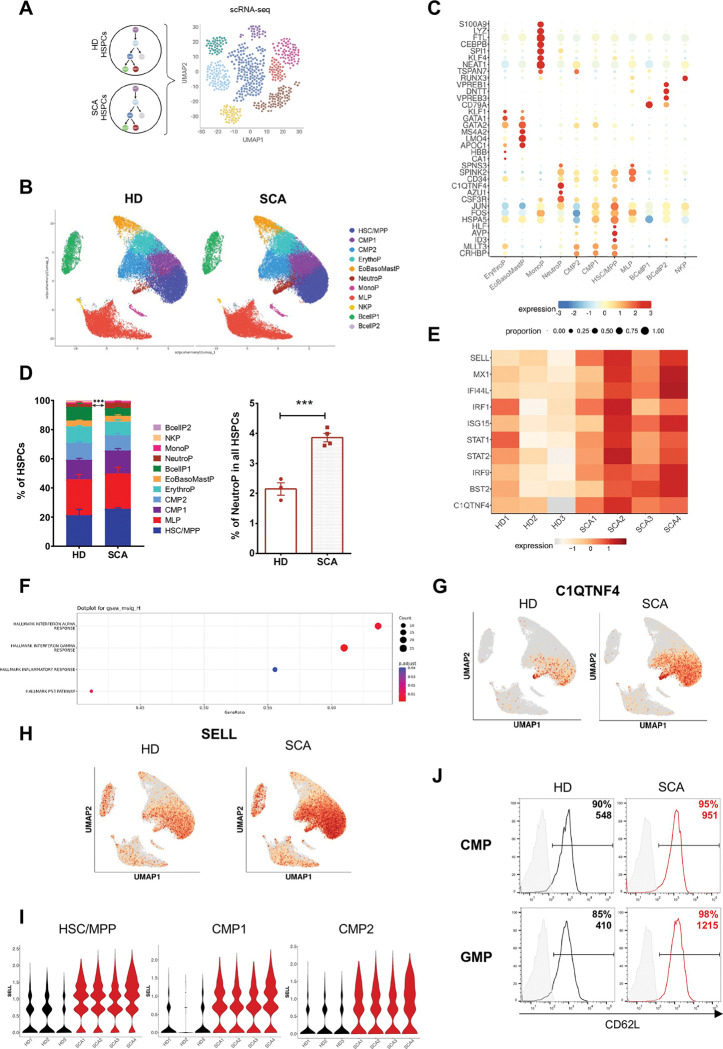
scRNAseq shows a myeloid bias of SCA HSPCs with enhanced type I IFN in neutrophil cluster. **(A)** HSPCs CD34^+^ cells from HDs and patients with SCA were analyzed by scRNA seq. **(B)** Unsupervised clustering of 64.254 circulating CD34^+^ cells from HDs (n=3) and patients with SCA (n=4), represented as UMAP. Cluster annotation was made using SingleR and reference PB HSPCs signatures ^26^. HSC: Hematopoietic stem cell, MPP: MultiPotent Progenitor, CMP: Common Myeloid Progenitor, ErythroP: Erythrocyte Progenitor, NeutroP: Neutrophil Progenitor, MonoP: Monocyte Progenitor, EoBasoMastP: Eosinophil Basophil Mast cell Progenitor, MLP: Multipotent Lymphoid Progenitor, NKP: Natural Killer Progenitors, BcellP: B cell Progenitor. **(C)** Expression values of selected marker genes for each cluster of HSPC combining controls and patients. Circle color shows mean expression values and circle size represents the proportion of expressing cells per cluster. **(D)** Frequency of each cluster among all HSPCs in HDs and patients with SCA. Percentages are plotted as bar histograms. Among all populations, NeutroP is the only cluster statistically increased in SCA compared to control. Statistical analysis was performed using an unpaired t-test; ***p=0.0009. **(E)** Heatmap of top genes differentially expressed in the NeutroP cluster between SCA and HD groups. **(F)** GSEA of hallmark gene sets MSigDB pathways on 997 DEGs in NeutroP cluster between HD and patients with SCA. **(G)** UMAP plots of *C1QTNF4* expression in HDs and patients with SCA. **(H)** UMAP plots of *SELL* in HDs and patients with SCA. **(I)** Expression levels of *SELL* in HSC/MPP, CMP1, and CMP2 clusters in each HD and patients with SCA. Differential gene expression analysis was performed using a Wilcoxon test. HSC/MPP: adj.p value=0, CMP1: adj.p value=0, CMP2: adj.p value=1,26×10^−240^. **(J)** CD62L (L-selectin) expression in CMP (CD34^+^CD38^+^CD123^+^CD45RA^−^) and GMP (CD34^+^CD38^+^CD123^+^CD45RA^+^) between HDs and patients with SCA analyzed by flow cytometry (n=2). Percentages of positive cells and GMFI are indicated.

**Figure 3.**
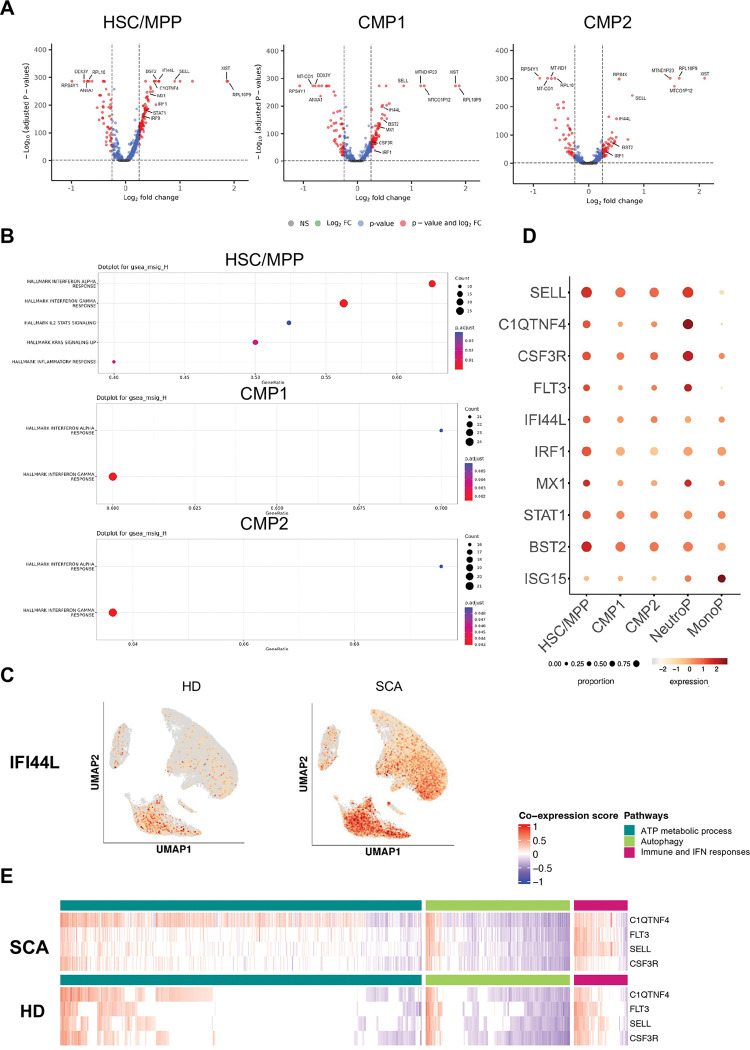
Activated type I IFN signaling is associated with myeloid signature in early HSPCs. **(A)** Volcano plots showing selected DEGs in HSC/MPP (left panel), CMP1 (middle panel), and CMP2 (right panel) between patients with SCA *vs* HDs. Genes in red are significantly differentially expressed with a log_2_ fold change > 0.25. Genes in blue are significantly differentially expressed with a log_2_ fold change < 0.25. **(B)** GSEA of hallmark gene sets MSigDB pathways on 1768 DEGs in HSC/MPP cluster (upper panel), 1195 DEGs in CMP1 cluster (middle panel) and 637 DEGs in CMP2 cluster (bottom panel) between HDs and patients with SCA. **(C)** UMAP plots of *IFI44L* expression in HDs and patients with SCA. **(D)** Plot showing the evolution of type I IFN and myeloid-specific gene expression across HSC/MPP, CMP1, CMP2, NeutroP and MonoP. Circle color shows mean expression values and circle size represents the proportion of expressing cells per cluster. **(E)** CS-CORE estimates in myeloid progenitors from SCA and HDs. We performed a differential co-expression analysis on top 5000 highly expressed genes in HSCs/MPPs, CMP1, CMP2, NeutroP and MonoP clusters and obtained modules of genes related to myeloid program (C1QTNF4, FLT3, SELL, CSF3R) in SCA and HDs. Positive scores indicate positive co-expression, while negative scores show absence of correlation.

Based on the previous findings, we hypothesized that enhanced myelopoiesis driven by an uncharacterized cytokine pathway could underlie leukocytosis in SCA. Therefore, we elected to characterize SCA HSPCs at the transcriptomic level. To this end, we performed 10x single-cell transcriptomic analysis on 64,264 circulating CD34^+^ cells isolated from 3 HDs and 4 untreated patients with SCA (Figure 2A). Using the batch corrected Principal component analysis (PCA) from Harmony, unsupervised Louvain clustering was performed using a range of values for the resolution parameter. The optimal resolution, plotted on the UMAP space, revealed 11 clusters, separating populations of HSPCs from both HD and SCA groups (Figure 2B). Hematopoietic cell identities were assigned to each cell cluster by comparing cluster-specific genes with a reported lineage signature gene list of PB HSPCs (Figure 2C, Table S2) ^26^. In the classical model of human hematopoiesis, the bulk of Granulocyte-Monocyte Progenitors (GMPs), characterized by CD45RA and CD123 expression, includes both granulocyte and monocyte progenitors. However, our scRNA analyses further resolved these populations into several distinct clusters (NeutroP, MonoP, EoBasoMastP). Each cluster was significantly enriched for specific lineage transcriptomic signature, with the exception of Common Myeloid Progenitor (CMPs) that might contain divergent myeloid fates, which were divided across two clusters (CMP1 and CMP2). Differentially expressed genes (DEGs) analysis between CMP1 and CMP2 revealed an upregulation of *JUN* and *FOS* genes, members of the AP-1 complex involved in monocytic differentiation, in CMP1 (Figure S1) ^27^. Next, we compared the proportion of each cluster in all HSPCs between patients with SCA and HDs. Interestingly, the percentage of neutrophil progenitors (NeutroP) was significantly higher in the SCA group (p<0.001), while no quantitative difference was observed in other HSPC clusters, including erythroid progenitors (Figure 2D). To further explore the transcriptional changes within the NeutroP cluster, we selected the top genes that were most highly expressed in SCA NeutroP compared to HDs. Notably, NeutroP from patients with SCA showed increased expression of Interferon Related Genes (*ISG15*, *IFI44L, MX1, IRF9)* and genes associated with Inflammatory response *(SELL and C1QTNF4)* in SCA NeutroP (Figure 3E, Table S3). Gene set enrichment analysis (GSEA) of hallmark pathways consistently highlighted activation of the interferon (IFN) signaling pathways and inflammatory response in SCA NeutroP (Figure 2F). Of particular interest, *C1QTNF4* gene, an effective marker of the neutrophil lineage ^28^, was highly expressed in the SCA NeutroP cluster and, unexpectedly upregulated in the HSC/MPP cluster, indicating an early neutrophilic commitment in SCA HSPCs (Figure 2G). Additionally, *SELL,* which encodes L-selectin (CD62L), a potential marker for myeloid-biased HSPCs ^29^, was upregulated in SCA HSPCs, with high expression in HSCs/MPPs, CMP1 and CMP2 and minimal expression in EryP and EoBasoMastP clusters, consistent with a myeloid bias in SCA HSPCs (Figure 2H-I). Flow cytometry analyses further confirmed a notable increase in CD62L expression in SCA cells, showing a two-fold and three-fold increase in CMP and GMP cells, respectively, when compared to HD cells (Figure 2J). Collectively, these data identify a myeloid bias of SCA HSPCs with enhanced type I IFN pathway activation in the NeutroP cluster.

### Activated type I IFN signaling is associated with myeloid signature in early HSPCs

To characterize the gene-regulatory networks underlying the myeloid bias in SCA, we further explored the expression program of early hematopoietic progenitors including HSC/MPPs, CMP1 and CMP2. As shown in Figure 3A, DEG analysis identified distinct patterns of upregulated genes in the HSC/MPP, CMP1 and CMP2 clusters from SCA HSPCs (Figure 3A, Table S3). The first expression pattern was enriched with type I and II IFN signaling and STAT activation-related inflammatory signaling. This group includes downstream effectors of IFN receptors such as *IFI44L*, *BST2*, *IRF1* and *STAT1*. The second expression pattern corresponds to upregulated myeloid-associated genes *SELL* and *C1QTNF4*. Consistent with these findings, IFN signaling pathways were significantly enriched in HSC/MPP, CMP1 and CMP2 clusters from SCA HSPCs compared to HDs (Figure 3B). Notably, IFN pathway activation, reflected by the upregulation of the *IFI44L* gene, was observed across all SCA HSPCs clusters including lymphoid and myeloid lineages (Figure 3C). The concomitant upregulation of both the myeloid program (*SELL, C1QTNF4 and CSF3R*) and IFN signaling in early HSPCs (HSCs/MPPs, CMP1, and CMP2 clusters) and myeloid-committed progenitors (NeutroP and MonoP clusters) indicates a continuous transcriptional co-program within SCA HSPCs (Figure 3D). This suggests a co-expression relationship between IFN signaling and myeloid bias in SCA HSPCs. Co-expression networks can be integrated to further understand this relationship. We then applied the CS-CORE statistical approach in 5,000 hub genes from HSCs/MPPs, CMP1, CMP2, NeutroP and MonoP clusters to compare key network modules in SCA versus HD HSPCs. We extracted co-expressed gene modules by applying WGCNA ^30^ on significantly co-expressed genes, which were then evaluated using Gene Ontology enrichment analysis. Finally, we constructed the differential co-expression modules driving the myeloid program (*SELL*, *CSF3R*, *FLT3* and *C1QTNF4*) between SCA and HD. Three modules related to myeloid program strongly enriched for GO terms differ between SCA and HD (Figure 3E, Table S4). Consistent with previous results, immune response including type I IFN signaling pathway was strongly co-expressed with *SELL*, *C1QTNF4* and *CSF3R* genes in SCA HSPCs compared to HDs (Figure 3E). In addition, GO analysis highlighted a significant enrichment of genes associated with critical metabolic pathways including ATP metabolic process as well as regulation of autophagy.

Collectively, these data highlight a continuous transcriptional myeloid program within SCA HSPCs, from the most immature clusters to committed myeloid progenitors, likely driven by IFN-I pathway activation.

### Phenotypic and functional characterization of SCA HSPCs confirm the increased commitment towards myeloid lineage.

**Figure 4.**
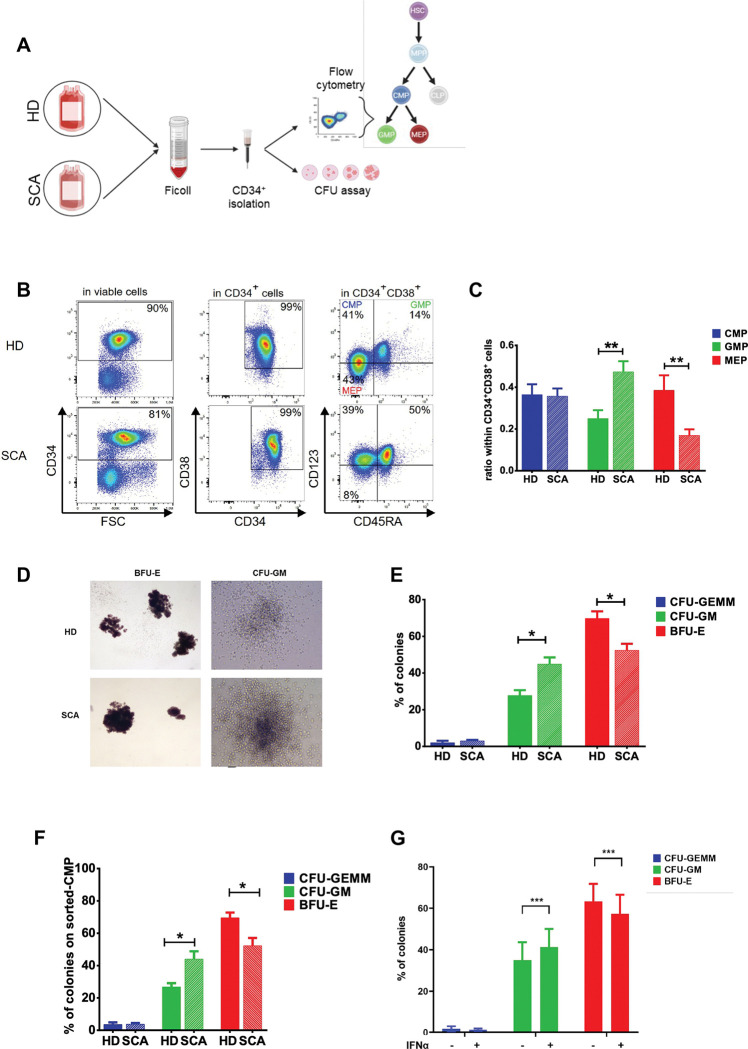
Circulating HSPCs from patients with SCA preferentially commit to the myeloid lineage. **(A)** Schematic representation of CD34^+^ cells characterization: CD34^+^ cells from HDs and patients with SCA were isolated after Ficoll gradient and magnetic separation. Then, cells were either used from CMP, MEP and GMP analysis by flow cytometry or plated for colony forming assay. **(B)** Circulating CD34^+^ cells from HDs and patients with SCA were analyzed with flow cytometry using CD34, CD38, CD123, and CD45RA markers. Representative plots show the percentages of CD34^+^ cells, CD34^+^CD38^+^ cells, and myeloid progenitors (CMP, GMP, GMP) for HDs and patients with SCA. **(C)** Ratio of CMP, GMP, and MEP within the number of CD34^+^CD38^+^ cells for HDs (n=6) and patients with SCA (n=10). Statistical analysis was performed using a Mann-Whitney test; MEP: **p=0.0042, GMP: **p=0.0075. **(D)** Circulating CD34^+^ cells from HDs and patients with SCA were plated in Methocult^™^ H4435 and grown for 14 days. Representative picture of BFU-E and CFU-GM. **(E)** Proportions of CFU-GEMM, CFU-GM, and BFU-E colonies in HDs (n=4) and patients with SCA (n=6) are plotted in bar histograms. Statistical analysis was performed using a Mann-Whitney test; CFU-GM: *p=0.0190, BFU-E: *p=0.0286. **(F)** CMP from HDs (n=3) and patients with SCA (n=4) were FACS-sorted on the basis of CD34, CD38, CD123, and CD45RA and grown in Methocult^™^H4435. Histograms represent the proportion of CFU-GEMM, CFU-GM, and BFU-E colonies at day 14. Statistical analysis was performed using a Mann-Whitney test; *p<0.05. **(G)** Circulating CD34^+^ cells from HDs (n=2) were treated overnight with or without IFN (500U/mL) and then plated in Methocult^™^ SFH4436 and grown for 14 days. The number of CFU-GEMM, CFU-GM, and BFU-E are shown. Statistical analysis was performed using a Mann-Whitney test; *p<0.05.

To further validate that leukocytosis is associated with abnormal HSPC myelopoiesis in SCA, we investigated the phenotypic composition of erythroid and myeloid progenitors isolated from the PB of untreated patients with SCA (Figure 4A). While the proportion of the Common Myeloid Progenitor (CMP: CD34^+^CD38^+^CD123^+^CD45RA^−^) within CD34^+^CD38^+^ cells was similar between HDs and patients with SCA, we observed a significant shift in the balance between Granulocyte-Monocyte Progenitors (GMP: CD34^+^CD38^+^CD123^+^CD45RA^+^), and Megakaryocyte-Erythrocyte progenitors (MEP: CD34^+^CD38^+^CD123^−^CD45RA^−^) (Figure 4B-C). There was a 1.9-fold increase in GMPs (p<0.01) and a 2.3-fold decrease in MEPs (p<0.01) in patients with SCA compared to HDs (Figure 4C). Next, we functionally explored the commitment of SCA HSPCs toward the myeloid and erythroid lineages by performing colony-forming assays in methylcellulose (Figure 4A). Interestingly, SCA HSPCs produced more CFU-granulocyte-macrophage (CFU-GM) colonies while the burst-forming unit erythroid progenitors (BFU-E) were significantly reduced compared to HD HSPCs (Figure 4D-E) (p<0.05). To further characterize the myeloid-biased HSPCs, myeloid progenitors including CMPs and GMPs were sorted and subjected to colony-forming assays. CMPs from patients with SCA exhibited a significant increase in CFU-GM colonies and a decrease in BFU-E colonies compared to HDs (p<0.05) (Figure 4F). In contrast, GMPs generated exclusively myeloid colonies in both groups (Figure S2). To further validate the role of IFN-I signaling in lineage specification, we treated HSPCs from HDs with IFNα and assessed their differentiation using CFU assays. Consistent with the previous finding, IFNα treatment significantly increased the proportion of myeloid colonies (Figure 4G), confirming the implication of IFN-I in the skewing of HSPCs towards the myeloid lineage. Altogether, these findings confirm an increased myeloid commitment of circulating HSPCs in SCA.

### *Ex vivo* granulopoiesis of HSPCs from patients with SCA showed unexpected monocytic differentiation.

**Figure 5.**
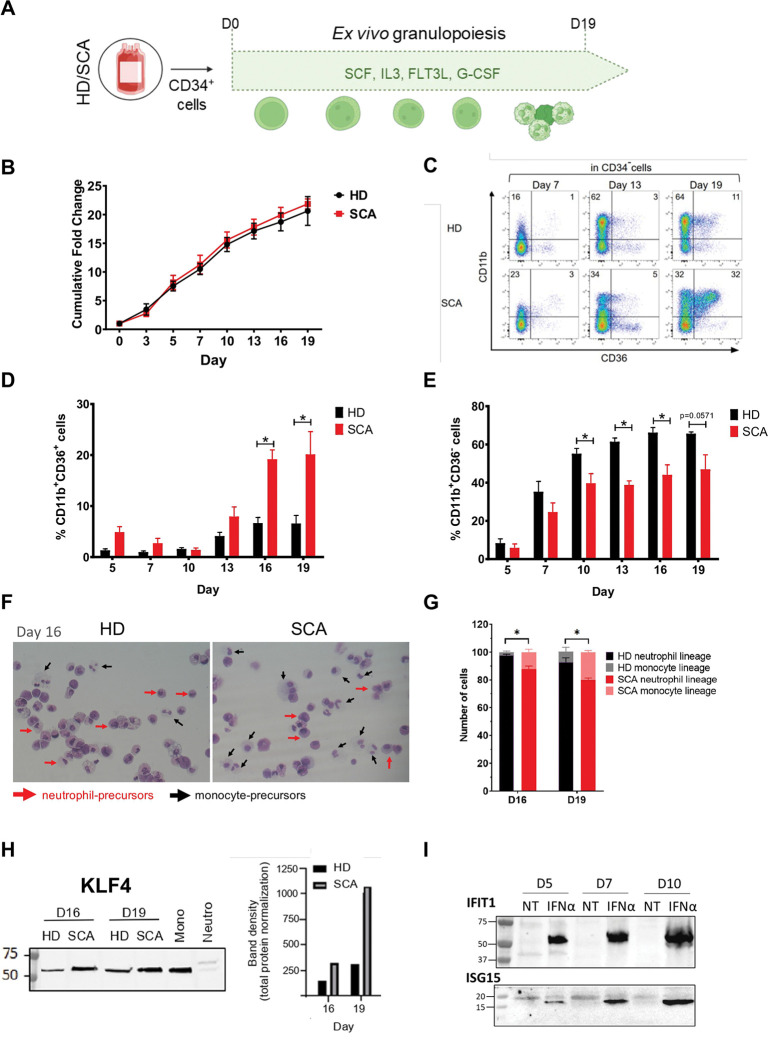
Unexpected increase of monocyte-lineage cells in *ex vivo* granulopoiesis from patients with SCA. **(A)** Representative protocol of *ex vivo* granulopoiesis: circulating CD34^+^ cells from HDs and patients with SCA were cultured with SCF, IL3, G-CSF and Flt3L for 19 days. **(B)** Growth curves are shown as cumulative fold change of myeloid cells from HD (n=5) and patients with SCA (n=4). **(C)** Myeloid maturation was monitored with CD36 and CD11b expression within CD34^−^ cells at several days of the differentiation. CD11b^+^CD36^−^ population represents neutrophil-lineage cells while CD11b^+^CD36^+^ subset is monocyte-lineage cells. **(D)** Percentages of CD11b^+^CD36^+^ and CD11b^+^CD36^−^ cells in HD (n=5; black) and patients with SCA (n=4; red) were plotted in bar histograms at days 5, 7, 10, 13, 16, and 19. Statistical analyses were performed using Mann-Whitney test: *p<0.05. **(E)** Representative picture of MGG staining of cells isolated from SCA and HD *ex vivo* granulopoiesis on day 16. Black and red arrows respectively indicate monocyte and neutrophil lineage cells. **(F)** Count of MGG-stained monocyte and neutrophil lineage cells from HD and SCA ex vivo granulopoiesis at day 16 and 19 (n=3). Statistical analysis was performed using unpaired t-test; *p<0,05. **(H)** Representative western blot of KLF4 expression at days 16, and 19 of HD and SCA *ex vivo* granulopoiesis, as well as in circulating HD monocytes and HD neutrophils. KLF4 expression was normalized based on total protein in each sample. Mono: monocytes HD; Neutro: neutrophils HD. **(I)** Circulating CD34^+^ cells from patients with SCA were cultured with SCF, IL3, G-CSF, Flt3L ± IFN-α (1ng/ml) for 19 days. Western blot of IFIT1 and ISG15 were performed on day 5, 7 and 10 of the differentiation.

To better characterize the myeloid features of these progenitors, we performed *ex vivo* granulopoiesis using CD34^+^ HSPCs isolated from HDs (n=5) and patients with SCA (n=4) cultured in the presence of G-CSF for 19 days (Figure 5A). Our results showed a similar granulocytic proliferation between HDs and patients with SCA (Figure 5B). In addition, Annexin V and Sytox Blue staining revealed similar percentages of apoptotic and necrotic cells throughout the differentiation (Figure S3). Similarly, cell cycle assay showed no difference between HDs and patients (Figure S4). Next, we investigated granulocytic maturation by analyzing the loss of HSPCs’ marker CD34 and the increase of CD11b myeloid protein expression. CD36 expression was also assessed to specifically distinguish CD11b^+^CD36^+^ monocytic-lineage cells from CD11b^+^CD36^−^ neutrophil precursors. Our results showed that CD34^+^ progenitors from HDs and patients with SCA exhibited a progressive loss of CD34 marker at the same rate in the first 13 days (Figure S5). However, patients with SCA displayed an unexpected differentiation of HSPCs towards the monocytic lineage, as shown by the significant increase of CD11b^+^CD36^+^ population by day 16 (p<0.05) and day 19 (p <0.05) of differentiation (Figure 5C-D). Specifically, within the CD34^−^ subset, only 6±2% of HD cells were CD11b^+^CD36^+^ while this population represented 20±5% of cells in the SCA group at day 19 (Figure 5D). This markedly increased monocytic differentiation was associated with a downregulation of granulocytic maturation, since from day 10 of the differentiation, CD11b^+^CD36^−^ cells were 1.6-fold less present in SCA differentiation than in HD (Figure 5E). The unexpected monocytic differentiation of SCA HSPCs in the presence of G-CSF was also confirmed by MGG staining (Figure 5F-G). Furthermore, Klf4, an essential transcription factor for monocyte differentiation ^31^, was upregulated at day 16 and 19 of SCA *ex vivo* granulopoiesis supporting the significant increase of monocyte-lineage cells in SCA (Figure 5H). Finally, we investigated the activation of IFN-I pathway during SCA terminal myeloid differentiation. While IFN response proteins ISG15 and IFIT1 were upregulated in IFNα-treated SCA CD34^+^, no basal activation of these proteins was found in untreated SCA CD34+ from day 5 to day 10 of myeloid differentiation (Figure 5I), suggesting that IFN signaling in CD34^+^ is not maintained during their myeloid differentiation. Together, these data indicate that a subset of HSPCs from patients with SCA are primed toward the monocytic lineage in the presence of the G-CSF.

### G-CSF induced monocytic differentiation of early myeloid progenitors in SCA

**Figure 6.**
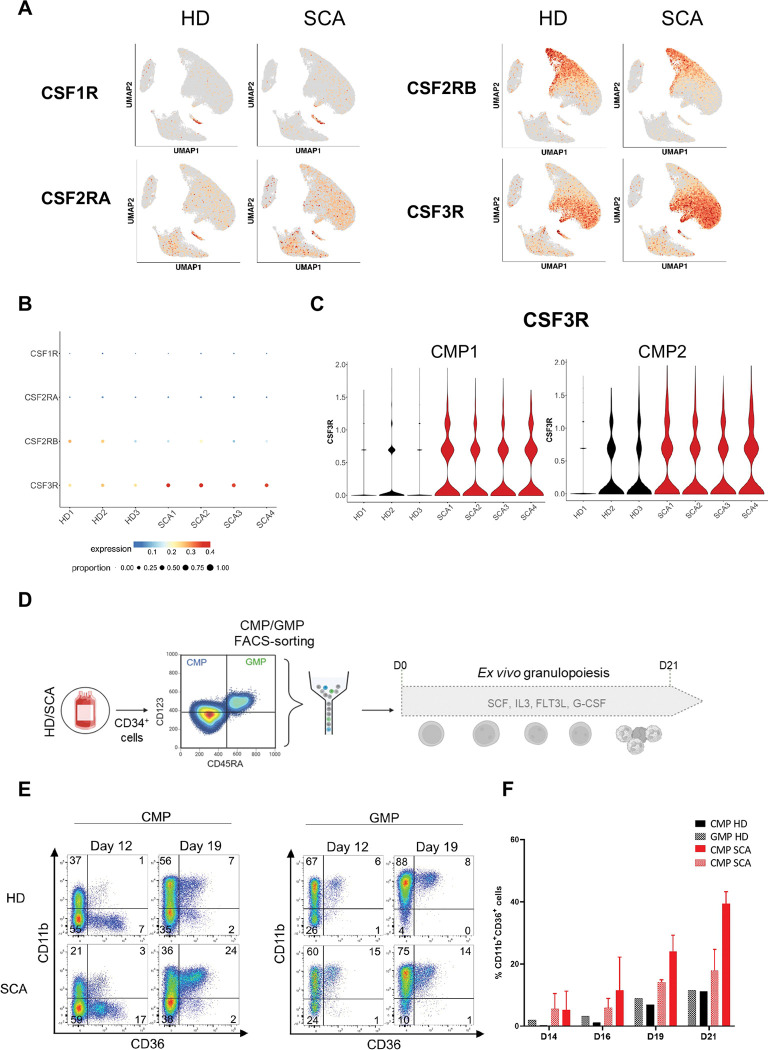
G-CSF induced monocytic differentiation of early myeloid progenitors in SCA. **(A)** UMAP plots of *CSF1R, CSF2RA, CSF2RB*, and *CSF3R* expression in HDs and patients with SCA. **(B)** Dotplot showing the expression of *CSF1R, CSF2RA, CSF2RB*, and *CSF3R* in all cells of scRNAseq. Circle color shows mean expression values and circle size represents the proportion of expressing cells per sample. Among these genes, *CSF3R* is the only significant one (Wilcoxon test). **(C)** Expression levels of *CSF3R* in CMP1, and CMP2 clusters in each HD and patients with SCA. Differential gene expression analysis was performed using a Wilcoxon test. CMP1: adj.p value=4,69×10^−76^, CMP2: adj.p value=7,18×10^−29^. **(D)** Sorted-CMP and GMP isolated from the peripheral blood of HDs and patients with SCA were cultured with SCF, IL3, G-CSF and Flt3L for 21 days. **(E)** Myeloid maturation was monitored with CD36 and CD11b expression within CD34^−^ cells at days 12 and 19. CD11b^+^CD36^−^ population represents neutrophil-lineage cells while CD11b^+^CD36^+^ subset is monocyte-lineage cells. **(F)** Percentages of CD11b^+^CD36^+^ and CD11b^+^CD36^−^ cells in CD34^−^ cells from sorted CMP and GMP in HD (n=1; black) and patients with SCA (n=3; red) are plotted in bar histograms at days 14, 16, 19 and 21.

M-CSFR (*CSF1R*), GM-CSFR (*CSF2RA* and *CSF2RB*), and G-CSFR (*CSF3R*) are important receptors for monocytic and granulocytic colony-stimulating factors. To further understand the G-CSF-induced monocytic differentiation of HSPC subset in SCA, we compared the expression levels of these receptors in HSPCs. Notably, reanalysis of the scRNA data revealed an overexpression of *CSF3R* in SCA HSPCs compared to HDs, while no significant changes were observed in *CSF2RA*, *CSF2RB* and *CSF1R* (Figure 6A-B, Table S3). Specifically, *CSF3R* is upregulated in early myeloid progenitors from patients with SCA including the CMP1 and CMP2 clusters (Figure 3A and 6C). Finally, we evaluated the ability of G-CSF to induce monocytic differentiation of bulk GMPs and bulk CMPs from HDs and patients with SCA. To this end, we sorted populations of CMPs and GMPs and performed *ex vivo* differentiation in the presence of G-CSF (Figure 6D). Unexpectedly, although neutrophils and monocytes originate from the GMPs, we observed that GMPs gave rise to few monocytic cells (CD11b^+^CD36^+^) in both HDs and SCA cells (Figure 6E). Indeed, enhanced differentiation potential towards monocytic lineage was observed exclusively in CMPs from patients with SCA (Figure 6E-F), consistent with the upregulation of *CSF3R* in CMP1 and CMP2 from scRNA data. Together, these results suggested an alteration of the G-CSF pathway in early HSPCs and myeloid bias of patients with SCA, in particular towards the monocytic lineage.

### Hydroxyurea attenuates the activation of IFN-I signaling pathway in mature neutrophils from patients with SCA.

**Figure 7.**
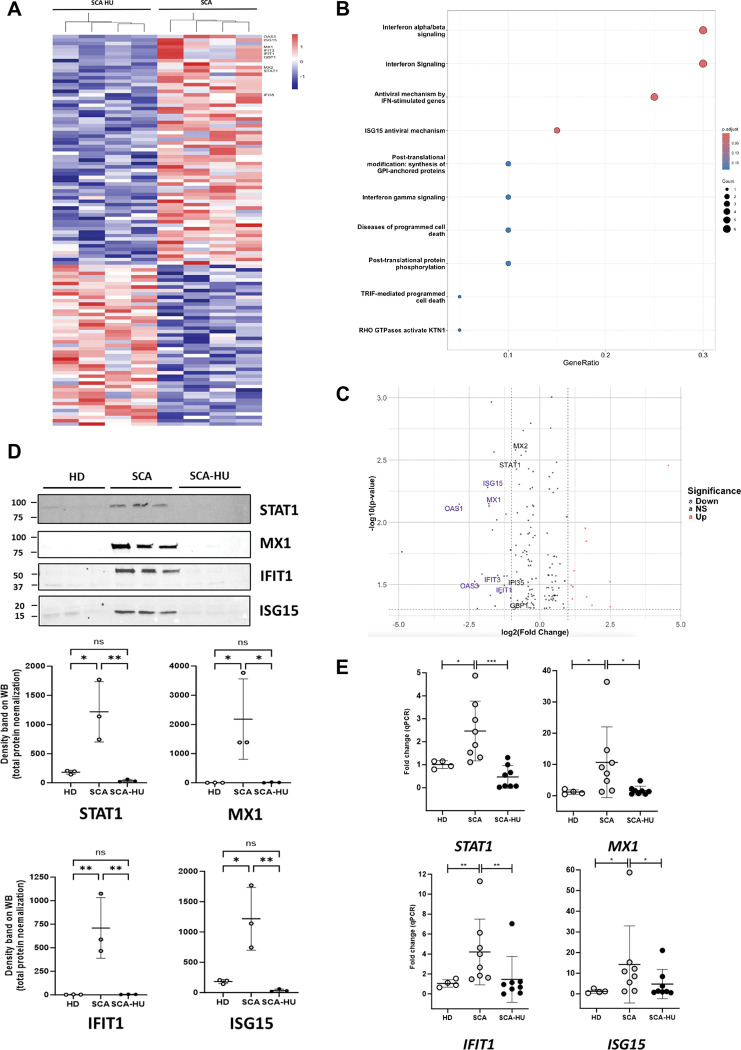
Hydroxyurea decreases IFN signature in mature neutrophils. (**A)** Cluster analysis of proteins differentially expressed in neutrophils from patients with SCA untreated (SCA, n=3) and treated with Hydroxyurea (SCA-HU, n=3). 151 proteins with significantly different expression values (p value<0.05 and fold change>0.3) were selected, their LFQ values were z score transformed and analyzed by Euclidian clustering. **(B)** Cluster analysis of differentially expressed proteins with the “cellular response to type I interferon” Reactom annotation. **(C)** Volcano plots showing selected DEGs in mature neutrophils between patients with SCA treated with HU*vs* untreated. **(D)**Expression of Interferon Signaling Proteins (STAT1, MX1, IFIT1, ISG15) by western blots in the circulating neutrophils from healthy donors (HD), patients with SCA untreated (SCA) and treated with Hydroxyurea (SCA-HU). The western blots are represented as the ratio of the density of the specific band on the total protein in each sample quantified on stain free gel. Statistical analyses were performed using One-Way Anova. **(E)** qPCR of STAT1, MX1, IFIT1 and MX1 gene expression in circulating neutrophils from HD, patients with SCA untreated (SCA) and treated with Hydroxyurea (SCA-HU). Expression was normalized using GAPDH. Statistical analyses were performed using One-Way Anova.

The main current treatment used to alleviate the symptoms of SCA is HU. In addition to its well-known effect of increasing fetal hemoglobin (HbF) levels in patients with SCA, HU also reduces neutrophil and monocyte counts, thereby helping to mitigate chronic inflammation. To investigate the effect of HU on the mature neutrophils, we performed a proteomic analysis of circulating neutrophils from age- and sex-matched patients with SCA, either treated or untreated with HU. After mass spectrometry analysis, 4366 proteins were reliably quantified. As previously reported by our team ^42^, neutrophils from untreated patients with SCA show a marked overactivation of type IFN-I signaling pathway. One hundred fifty-one proteins were significantly differentially expressed using a SCA/SCA-HU ratio > 1.3 or < 0.7. Among the sixty-seven proteins down-regulated in the SCA-HU group compared to the untreated SCA, we found a decrease in Interferon Related Protein’s expression such as OAS3, ISG15, MX1, IFIT3, IFIT1, GBP1, MX2, STAT1 and IFI35 (Figure 7A). 2D annotation enrichment test using Reactom annotation databases shows expression pattern enriched with IFN signaling, particularly type I-IFN pathways within a down-regulation of Interferon-related proteins expression in SCA-HU neutrophils compared to untreated SCA neutrophils (Figure 7B), confirmed by DEG analysis (Figure 7C). These results were confirmed by Western-blot analysis (Figure 7D), and qPCR (Figure 7E) of *STAT1*, *MX1*, *IFIT1* and *ISG15*, collectively indicating that HU attenuates the activation of IFN-I signaling pathway in mature neutrophils from patients with SCA.

## Discussion

SCA severity, marked by both acute events (such as VOCs and acute chest syndrome) and chronic complications from progressive organ damage, is closely tied to uncontrolled inflammation. This inflammation is driven by elevated levels of neutrophils and monocytes, which trigger the release of proinflammatory cytokines like TNF-α, IL-6, and IFN-α ^2,10^. Understanding the exact mechanisms behind leukocytosis is crucial for disrupting the vicious cycle of inflammation in SCA. As observed in other cohorts, we demonstrated that leukocytosis appeared very early in infants with SCA, within the first two years of life ^32^. While defects in hematopoiesis have long been suspected, they were never clearly demonstrated due to the lack of comprehensive characterization of CD34^+^ HSPCs from untreated patients with SCA ^12,33^. In this study, we combined transcriptomic, phenotypic, and functional approaches to reveal a myeloid-primed signature in circulating SCA HSPCs, which reflect enhanced myelopoiesis and contributes to the elevated neutrophil and monocyte counts. Using well-established experimental systems, we demonstrated that the myeloid program is activated early in SCA HSPCs, leading to an expanded NeutroP cluster observed in scRNA-seq analysis, which was further supported by an increased frequency of GMPs. G-CSF is considered as the master regulator of proliferation, differentiation, and survival of neutrophils ^34,35^. In SCA HSPCs, we have demonstrated that G-CSF stimulates the *ex vivo* expansion of not only neutrophils but also monocytes in the absence of inflammatory stimuli. In addition, our findings suggest that G-CSF induces monocytic differentiation not in classical progenitors like GMPs, but rather in early myeloid progenitors within the CMP population. Recent xenograft studies have shown that plerixafor-mobilized HSPCs from patients with SCA, when transplanted into immunodeficient mice, exhibit enhanced differentiation toward the myeloid lineage and generate more CD14^+^ cells compared to those from HDs ^19^. This finding is consistent with our *ex vivo* observation suggesting a transcriptional reprogramming of SCA HSPCs toward a myeloid fate. Atypical G-CSF-induced monocytosis has been reported in patients with leukemia ^36,37^. However, the exact role and origin of these monocytes have not been understood. In a recent study, Ikeda et al. find that immunoregulatory monocytes expand in response to G-CSF during emergency myelopoiesis^38^. Unlike classical monocytes that differentiate from monocyte-dendritic cell progenitors through the common monocyte progenitors, immunoregulatory monocytes differentiate from early neutrophil progenitors within the GMP population. Hence, in our data, G-CSF-induced monocytic differentiation is specific to SCA CMP, suggesting that emergency myelopoiesis is not involved in this process. Accordingly, G-CSF induces emergency myelopoiesis through upregulation of C/EBPβ transcription factor (*CEBPB*) ^39^, and our scRNA analyses showed normal expression of *CEBPB* in SCA and HDs HSPCs (data not shown). For these reasons, our observation that G-CSF-induced *ex vivo* monocytic differentiation of SCA CMP is likely to be associated with the upregulation of *CSF3R* in early SCA myeloid progenitors. Whether CSF3R^+^ cells within the CMP represent a multipotent progenitor or contain a small fraction of progenitors with a potential to differentiate into monocytes remains to be determined to confirm the alteration of the G-CSF-CSF3R axis in SCA hematopoiesis.

Recent transcriptomic and proteomic analyses have revealed elevated expression of IFN-stimulated genes (ISGs) in multiple immune cell populations from patients with SCA ^40–42^. Notably, in our previous study, we reported an abnormal activation of IFN-I pathway in neutrophils from SCA ^42^. Interestingly, in this work, we demonstrated that HU treatment reduces the IFN-I signaling in neutrophils from HU treated patients. This observation is in line with previous reports showing that HU significantly decreases absolute neutrophil counts and neutrophil-driven inflammation, which are critical contributors to vascular damage and chronic inflammation ^43,44^. Clinical and translational studies have shown that HU therapy not only reduces leukocytosis but also downregulates inflammatory cytokines such as TNF-α, IL-8, and soluble adhesion molecules, thereby lowering neutrophil activation and vascular adhesion ^45^. Importantly, this anti-inflammatory and cytoreductive effects extend to the bone marrow microenvironment, where HU has been reported to suppress excessive granulopoiesis and restore more balanced hematopoietic output ^46^. Together, these findings suggest that HU alleviates aberrant IFN-I signaling in neutrophils likely by reducing their numbers and/or by reprogramming their activation state, ultimately contributing to improved hematopoietic homeostasis in SCA.

Emerging data also suggest that IFN-I signaling extends its influence on SCA hematopoiesis. Chronic IFN-I exposure is known to affect HSPC function by promoting cell cycle entry, reducing self-renewal capacity, and skewing lineage commitment ^22,47–49^. In the current study, we demonstrate that this pathway is also activated in SCA HSPCs and, more importantly, is significantly associated with the early myeloid programming within the HSC/MPP and CMP clusters. Consistently, a recent study reported an association between hyperactivation of IFN-I pathway and the myeloid bias in CD34^+^ from bone marrow of two patients with SCA treated with the lentiviral vector (DREPAGLOBE) expressing a potent anti-sickling β-globin ^50^. These findings support a model wherein IFN-I signaling contributes to transcriptional reprogramming of SCA HSPCs, favoring myelopoiesis and contributing to the elevated myeloid output observed in SCA. Although recent studies indicate that type I IFNs can upregulate the expression of macrophage colony-stimulating factor receptor (M-CSFR) on GMP via STAT1 activation ^51^, its association with *CSF3R* expression in SCA remains to be proved.

In the Townes mouse model of SCA, the type I IFN pathway is constitutively activated and contributes to several pathophysiological processes. These include impaired erythropoiesis, likely through negative regulation of erythropoietin (EPO) and its receptor (EPOR) signaling, increased monocyte recruitment, enhanced erythrophagocytosis, and dysregulated antibody production ^40,52^. Hemolysis and the release of free heme have been suggested as key drivers of this IFN-I hyperactivation in this model ^53,54^. In addition, HbS has been shown to trigger monocyte activation via Toll-like receptor 4 (TLR4), promoting IFN-α release ^55^. Although Townes mice exhibit both leukocytosis, primarily due to elevated lymphocyte counts, and HSC exhaustion ^16^, the phenotype and feature of their HSPCs remains poorly documented. Notably, a myeloid bias has not been clearly demonstrated in these mice.

In conclusion, we have demonstrated that impaired myeloid fate decisions in early HSPCs from patients with SCA, along with an abnormal transcriptional program, contribute to leukocytosis and chronic inflammation in SCA. A limitation of this study is the use of CD34^+^ cells from peripheral blood rather than bone marrow. However, bone marrow-derived HSPC samples are almost exclusively available from patients with SCA treated with HU, a potent modulator of hematopoiesis. Although future studies are needed, the proposed link between the type I IFN pathway, G-CSF responsiveness, and the myeloid bias of circulating CD34^+^ cells provide important pathophysiological insights into their behavior post-engraftment in gene therapy settings as well as their involvement in the initiation and persistence of chronic inflammation in patients with SCA.

## Supplementary Material

Supplementary Files

This is a list of supplementary files associated with this preprint. Click to download.

• Suppmaterial.docx

• TableS2.xlsx

• TableS3.xlsx

• TableS4.xlsx

## Data Availability

ScRNAseq dataset are available on European Genome-Phenome Archive (EGA) database under the following number: EGAD50000001522.

## Key points

- Leukocytosis in SCA is associated with a skewing of HSPCs towards myeloid lineage and enhanced monocytic differentiation
- Type I interferon pathway and myeloid program are co-activated in early SCA HSPCs
